# Persistent Coronavirus Disease 2019 Headache Relieved with Sphenopalatine Ganglion Block

**DOI:** 10.5152/TJAR.2022.21318

**Published:** 2022-04-01

**Authors:** Danielle Levin, Martin Acquadro, Joseph Cerasuolo, Frederic J. Gerges

**Affiliations:** Department of Anaesthesia, St. Elizabeth’s Medical Center, Brighton, Boston, USA

**Keywords:** COVID-19, headache, outpatient anaesthesia, pain, sphenopalatine ganglion block

## Abstract

According to the World Health Organization, as of September 2021, there have been over 226.8 million people diagnosed with coronavirus disease 2019 and over 4.6 million deaths from this disease. Out of those who have survived the coronavirus disease 2019 infection, many individuals have symptoms that linger on. We would like to describe the first report of a patient with a 5-month history of a persistent coronavirus disease 2019 headache, which was finally successfully aborted with a single transnasal sphenopalatine ganglion block. A female in her early 50s presented to our pain clinic after suffering from a new, debilitating headache that began with the coronavirus disease 2019 illness and persisted for 5 months. Every evening the patient would experience a severe headache located deep inside/behind the left eye that would be resistant to analgesic medications. After 1 transnasal sphenopalatine ganglion block, the patient’s coronavirus disease 2019 headache was completely resolved.

## Main Points

To raise awareness of how coronavirus disease 2019 (COVID-19) can cause individuals to continue to suffer from persistent headaches even after the COVID-19 infection has resolved.

To describe the first report of successful treatment of an intractable 5-month COVID-19 headache with sphenopalatine ganglion block.

To share how to create an effective and inexpensive cotton-tipped applicator for a sphenopalatine ganglion block from supplies that are readily available in most medical facilities.

## Introduction

According to the World Health Organization, as of September 2021, there have been over 226.8 million people diagnosed with coronavirus disease 2019 (COVID-19) and over 4.6 million deaths from this disease.^1^ Out of those who have survived the COVID-19 infectious disease, many individuals have symptoms that persist, called “COVID long-haulers” or suffers of “long COVID.” Since the start of the global pandemic, headache has been reported as a frequent symptom of COVID-19, but it is now apparent that for some individuals, a disabling headache persists even after the resolution of COVID-19.^2-3^ Unfortunately, these COVID-19-related cephalgias are often resistant to analgesics.^4^ We would like to describe here the first report of a patient with a 5-month history of a COVID-19 headache that was successfully aborted with a single transnasal SPGB session.

## Case Presentation

In November 2020, a patient in her early 50s with a past medical history of hypertension, hyperlipidemia, chronic obstructive pulmonary disease, arthritis, depression, panic disorder, chronic back pain, and fibromyalgia was diagnosed with COVID-19 and experienced fatigue, sore throat, cough, shortness of breath, anosmia, fever, bilateral ear infections, and a new type of headache that she has never felt before. The patient was hospitalized for 5 days, and she was treated with remdesivir and steroids. By January 2021, most of the patient’s symptoms resolved, but she continued to experience fatigue, cough, shortness of breath, anosmia, and a new headache.

In April 2021, the patient came to our pain clinic in hopes of receiving help for her new headache. The patient described the headache as a severe, debilitating pain located deep inside/behind the left eye that would occur every evening. This pain would last through the evening, and sometimes, the patient would even wake up in the morning with it. The patient had tried ice, acetaminophen, and opioids, without significant relief. Regardless of her day-to-day activities, that same headache would occur daily since the COVID-19 disease, for 5 months. Along with this new headache, the patient did have nasal congestion but did not report other autonomic symptoms such as conjunctival injection or lacrimation.

Considering that the sphenopalatine ganglion block (SPGB) has previously been shown to relieve the pain of various etiologies,^5-10^ we offered this treatment option to this patient. Upon discussion of potential risks and benefits of the procedure, the patient elected to go ahead and signed the written informed consent for SPGB. 

**Figure 1.**
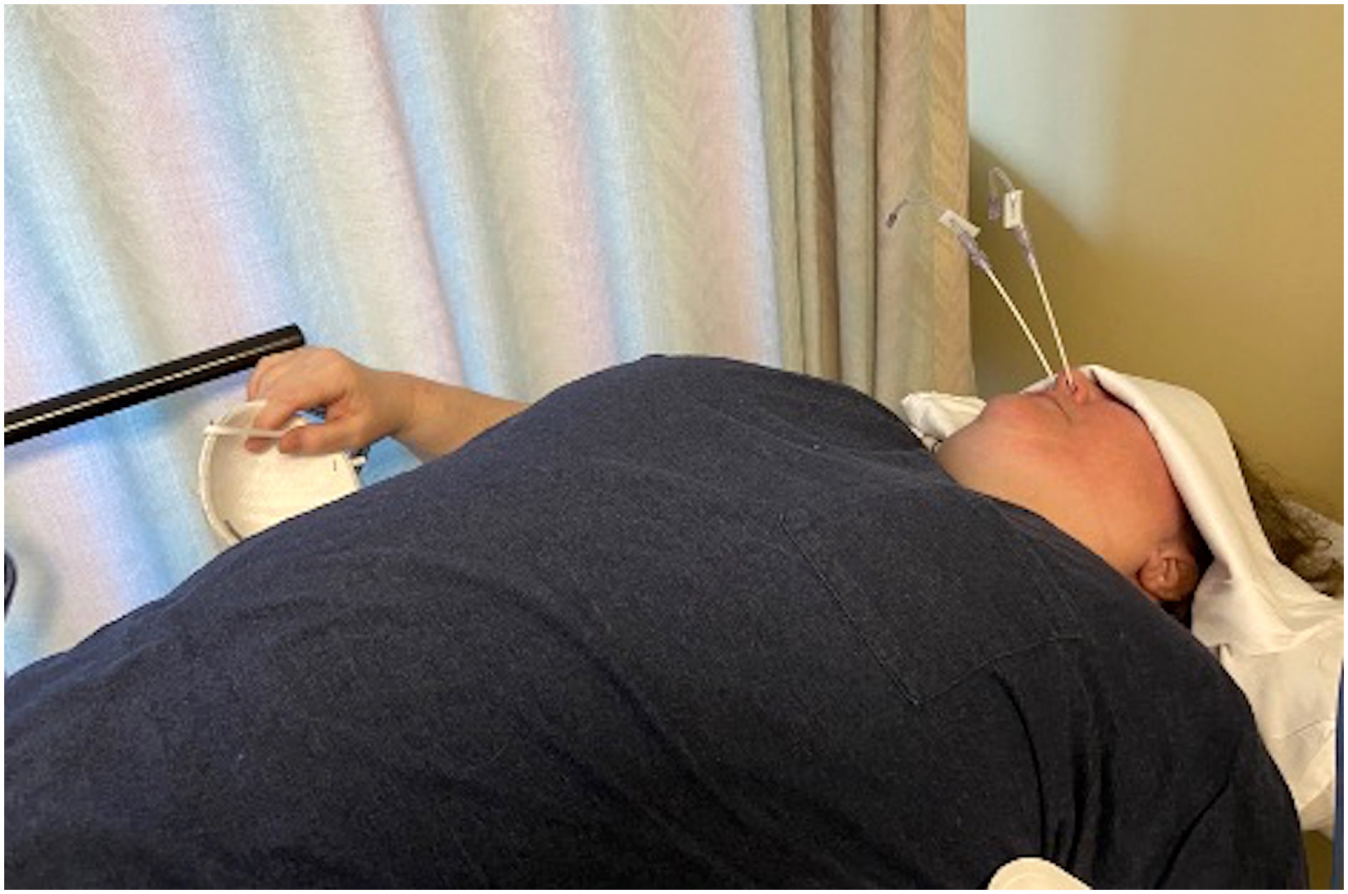
The patient receiving the transnasal sphenopalatine ganglion block via the cotton-tip applicators.

For the procedure, the patient was in a supine position with her chin-up. Long hollow cotton-tip applicators, dipped into lidocaine ointment USP, 5%, were placed atraumatically into both nasal sinuses. They were advanced until gentle resistance was met at the back of the nasopharynx. Lidocaine 4% was dripped drop by drop through the hollow cotton-tip applicators into each nostril until the patient felt the medication in the back of her throat (Figure 1). The cotton-tip applicators were left in place for a total of 15 minutes, and this was repeated a total of 3 times that day. A total of 4.75 mL of lidocaine 4% was administered in the right nostril in divided doses, and a total of 3.50 mL of lidocaine 4% was administered in the left nostril in divided doses.

The patient was followed up 1 month later, and she reported that since the SPGB treatment, the nightly headaches that she has had since her COVID-19 infection never returned. The patient provided written informed consent for publication of her case and her picture.

## Discussion

When the novel coronavirus named COVID-19 (caused by severe acute respiratory syndrome coronavirus 2) was initially discovered, and it was characterized by the manifestation of respiratory symptoms. However, with the progression of the COVID-19 pandemic, it has become apparent that the presentation of this disease varies and may adversely affect practically any organ in the human body. Reports of neurological findings are increasing rapidly and headache is among the top symptoms.^3^ Literature review shows that up to 71% of patients with the alpha-variant of COVID-19^11^ and most patients with the delta-variant of COVID-19 experience headaches.^12^

Like with many other concepts about the COVID-19 illness, it is currently unknown why people with COVID-19 have headaches. However, 1 theory of the COVID-19 headache pathophysiology is that the virus causes the human body to release pro-inflammatory mediators and cytokines. Various inflammatory mediators, such as nitric oxide, prostaglandin E2, interleukin 1, and nuclear factor-kB, result in neuroinflammation and trigger the activation of the perivascular trigeminal nerve endings, which can cause a severe headache.^3,13^

Inflammation is a normal response to a pathogenic infection, but it appears that individuals with noncommunicable chronic diseases, like our patient in the case presentation, have a pre-heightened inflammatory level.^14^ This can subject these patients to uncontrolled inflammation when infected with COVID-19. The loss of regulation on the release of pro-inflammatory cytokines (aka “cytokine storm”) could be the explanation behind the persistence of the COVID-19 headache.

So far, there is no established effective treatment to abort these COVID-19 headaches. Previously, it has been reported that 1 individual has been suffering from these intractable headaches for over 80 days.^15^ Unfortunately, our patient had been affected by this pain for even longer. 

Considering that the SPGB, first discovered in 1909, has been shown to relieve headaches of various etiologies,^7-10^ we offered this treatment to our patient. The sphenopalatine ganglion is a collection of nerve cells that is in close proximity to the trigeminal nerve. When lidocaine is administered drop by drop intranasally and then felt in the back of the throat during the conduction of the SPGB, the medication is able to seep into the sphenopalatine ganglion and anaesthetize it. This results in abruption of the nociceptive signaling and possibly decreases the neuroinflammatory process that is occurring in the perivascular trigeminal nerve endings. We theorize that through the SPGB, we were able to reduce the activation of the perivascular pain receptors that were provoked by the COVID-19 disease and stop our patient’s daily pain cycle.

## Conclusion

This is the first report of successful treatment of an intractable 5-month COVID-19 headache with SPGB using a cotton-tipped applicator. Although there are several commercially available devices, the SPGB can also be effectively performed by quickly and inexpensively creating cotton-tip applicators from supplies that are readily available in most medical centers. Our patient described this procedure as a “miracle treatment” since it broke her COVID-related headache cycle, and we hope this can help others who are also suffering from intractable COVID-19 headaches. 

### Informed Consent:

Written informed consent was obtained from the patient who agreed to take part in the study.

### Peer-review:

### Externally peer-reviewed.

### Author Contributions:

Concept – D.L., M.A., J.C., F.J.G.; Design – D.L., M.A., J.C., F.J.G.; Supervision – M.A., F.J.G.; Resources – D.L., M.A., J.C., F.J.G.; Materials – D.L., M.A., J.C., F.J.G.; Data Collection and/or Processing – D.L., M.A., J.C., F.J.G.; Analysis and/or Interpretation – D.L., M.A., J.C., F.J.G.; Literature Search – D.L., M.A., F.J.G.; Writing Manuscript – D.L., M.A., J.C., F.J.G.; Critical Review – D.L., M.A., J.C., F.J.G.

### Acknowledgments:

We would like to first thank the patient for allowing us to take care of her and for her permission that allows us to share her medical journey with the readers of this manuscript. We also express our sincere gratitude to the nurses, residents, and other staff for their help with taking care of this patient.

### Declaration of Interest:

The authors have no conflict of interest to declare.

### Funding:

The authors declared that this study has received no financial support.
